# Supplementary figures and images for: TDP-43 mutations link Amyotrophic Lateral Sclerosis with R-loop homeostasis and R loop-mediated DNA damage
Source: PLoS Genet. 2020 Dec 10;16(12):e1009260. doi: 10.1371/journal.pgen.1009260 (PMC7755276; doi:10.1371/journal.pgen.1009260)

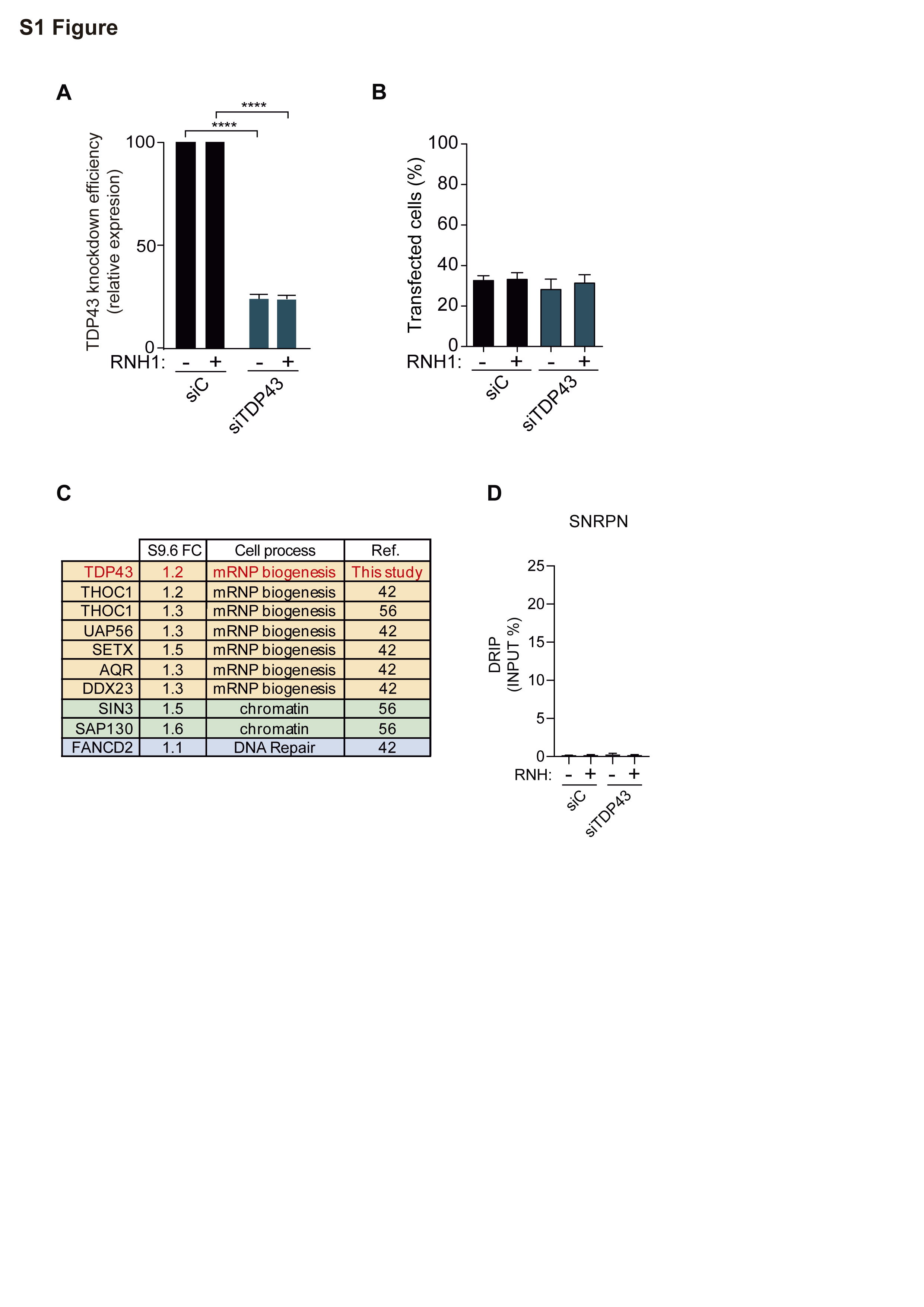

Supplement: S1 Fig — A) Relative TDP43 expression as measured by cDNA-qPCR in control (siC) and TDP-43-depleted (siTDP43) cells. Unpaired t test, two-tailed was done. B) Percentage of cells efficiently transfected (GFP+) overexpressing RNH1. C) Comparative table on S9.6 FC (fold change) between results presented in this study for TDP-43 and others reported for factors involved in R loop homeostasis in HeLa cells. Table shows the relative increase in S9.6 reactivity (S9.6 FC), the cell process in which the factor is involved and the reference. D) DRIP-qPCR at the SNRPN (negative control) gene in siC and siTDP-43-treated HeLa cells gDNA untreated (-) and treated (+) with RNH. Unpaired t test, one-tailed was done. For all cases: Blank, not significant; *, P < 0,05; **, P < 0,01; *** P < 0,001; ****, P < 0,0001. (TIF) [file pgen.1009260.s001.tif]

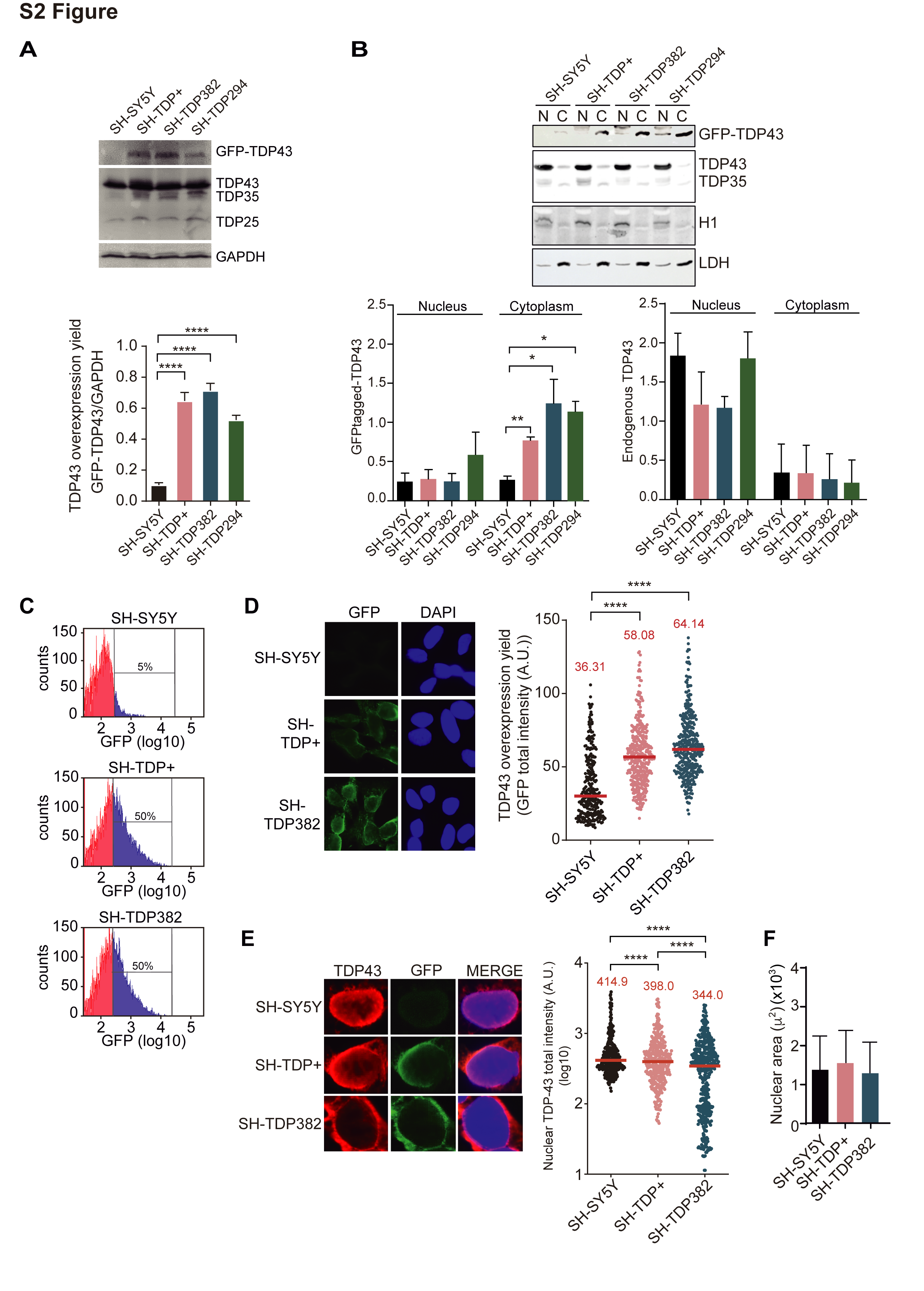

Supplement: S2 Fig — A) WB against GFP, TDP-43 and GAPDH in SH-SY5Y, SH-TDP+, SH-TDP382 and SH-TDP294. Relative quantifications of TDP43 overexpression rate, measured as GFP-TDP-43/GAPDH, are indicated in the histogram (n = 3). Unpaired t test, two-tailed was done. B) Western blot analysis of nuclear and cytoplasmic fractions of endogeneous TDP-43 and overexpressed TDP-43 forms fused to GFP. Mean+SEM (n = 3) are indicated in the histogram. *, P < 0,05; **, P < 0,01 (Unpaired t test, two-tailed). C) Flow cytometry analysis of TDP-43 overexpression. GFP intensity (GFP-TDP43) for each cell line is shown. Percentage of cell over the threshold are indicated. D) TDP-43 overexpression efficiency as measured by IF. Representative images are showed in the left part, while scatter plot in the right presents TDP43 overexpression rates, as measured by total GFP intensity (GFP-TDP43) in cell population. Median values are indicated. Scale bar: 25μm. Mann-Whitney U test, two-tailed was done. E) IF using TDP-43 and GFP antibodies on SH-SY5Y, SH-TDP+ and SH-TDP382. TDP-43 nuclear abundances for each cell line, measured as total TDP43 intensity, are indicated in the scatter plot. Median values are indicated. Scale bar: 25μm. Mann-Whitney U test, two-tailed was done. F) Nuclear area in SH-SY5Y, SH-TDP+ and SH-TDP382. Mean+SEM (n = 3) are indicated in the histogram. Other details as in Fig 4 and S1 Fig. (TIF) [file pgen.1009260.s002.tif]

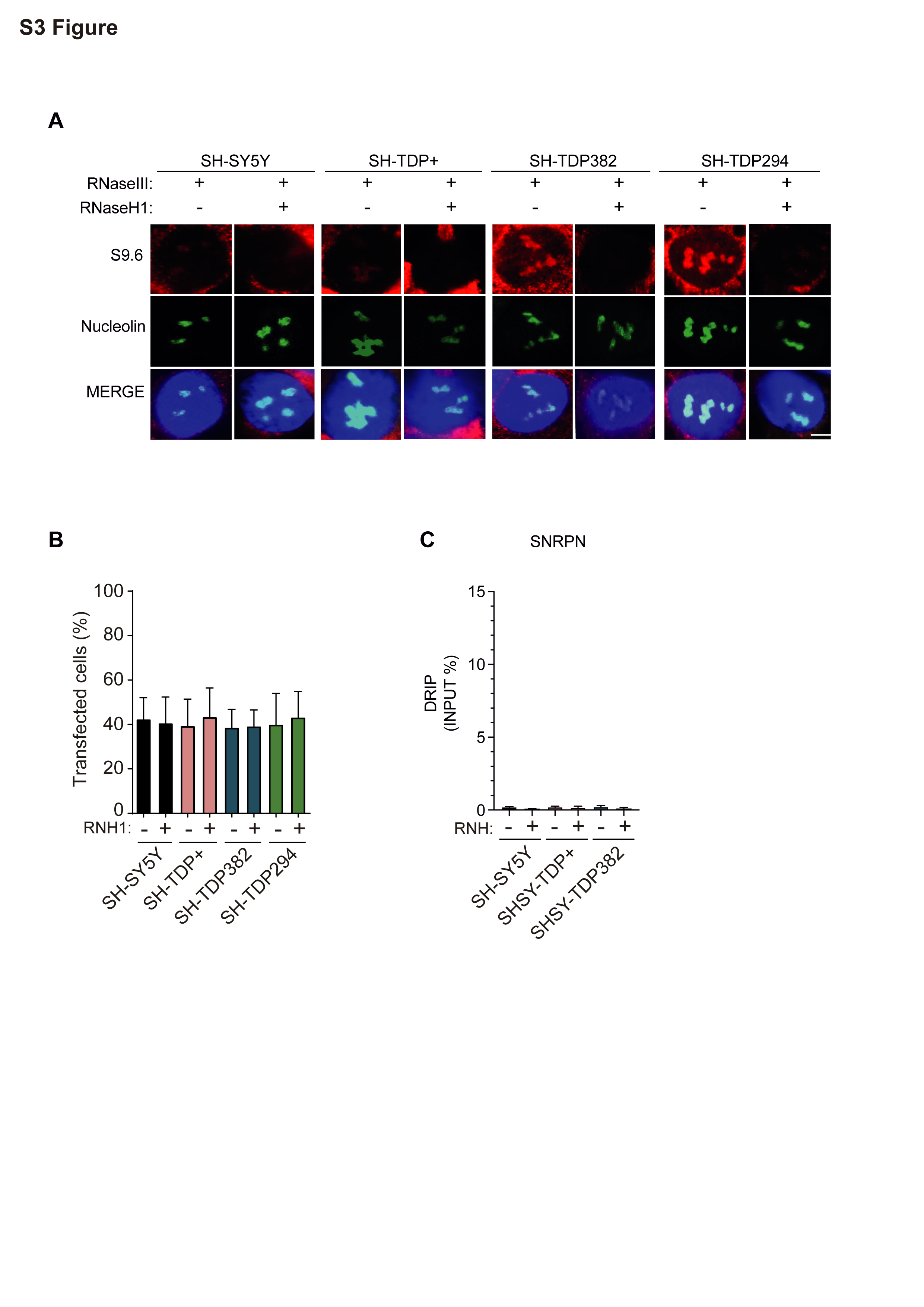

Supplement: S3 Fig — A) Representative images of S9.6 and nucleolin IFs in SH-SY5Y, SH-TDP+, SH-TDP382 and SH-TDP294. Scale bar: 25μm. B) Percentage of cells efficiently transfected (GFP+) overexpressing RNH1. Unpaired t test, two-tailed was done. C) DRIP-qPCR at the SNRPN (negative control) gene in SH-SY5Y, SH-TDP+ and SH-TDP382 cells gDNA untreated (-) and treated (+) with RNH. Other details as in Fig 4 and S1 Fig. (TIF) [file pgen.1009260.s003.tif]

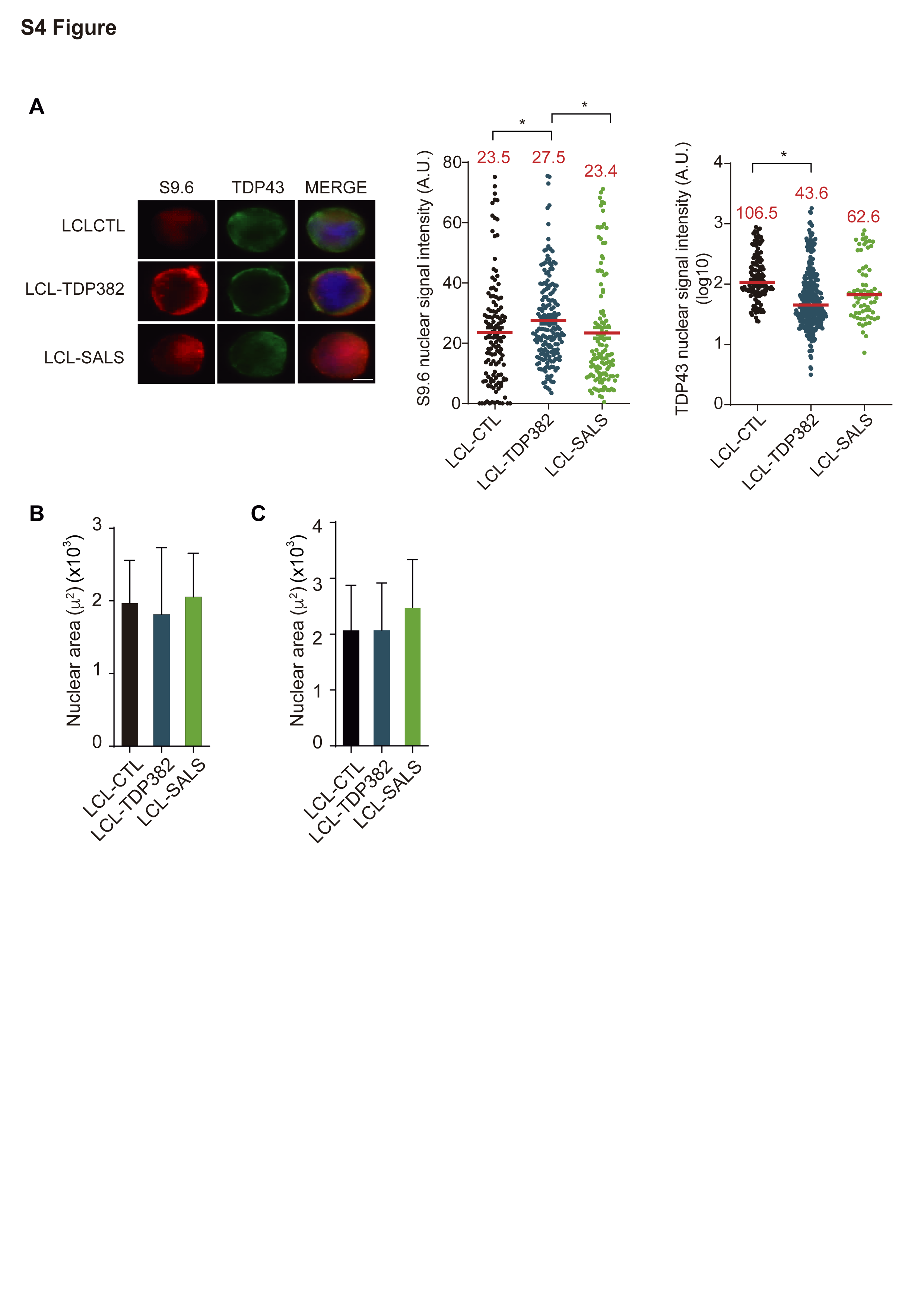

Supplement: S4 Fig — A) IF of LCL-CTL, LCL-TDP382, LCL-SALS using an anti-TDP-43 antibody and an anti-S9.6 antibody after paraformaldehyde fixation. The scatter plots show the increase of S9.6 signal intensity and the decrease in TDP43 nuclear content in SH-TDP382. Median values are indicated. Scale bar: 10μm. Mann-Whitney U test, two-tailed was done. Nuclear area in SH-SY5Y, SH-TDP+ and SH-TDP382 when fixed in paraformaldehyde (B) and methanol (C) are indicated. Median+SEM are indicated in the histogram. Unpaired t test, two-tailed was done. Other details as in Fig 6 and S1 Fig. (TIF) [file pgen.1009260.s004.tif]

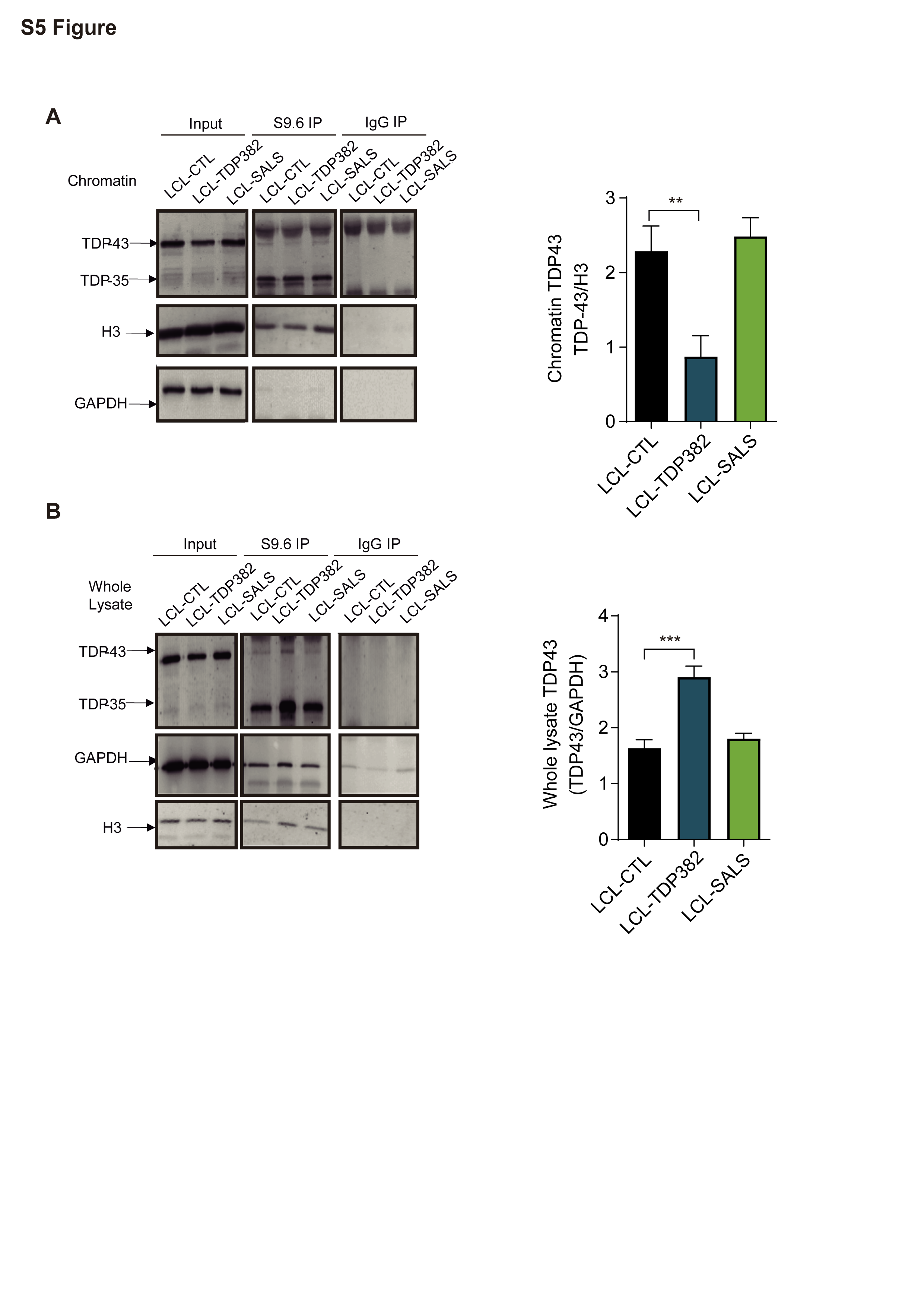

Supplement: S5 Fig — TDP-43 and TDP-35 strongly interact with S9.6 antibody in TDP-43 mut LCLs WL fraction. A) and B) CoIP between S9.6 and TDP-43 in chromatin of LCL-CTL, LCL-TDP382, LCL-SALS. Input, S9.6 IP and IgG IP of chromatin fraction were loaded on a 10% SDS-PAGE and then immunoblotted with TDP-43, H3 and GAPDH as nuclear and cytosolic loading control. S9.6 binding was tested by qPCR. Quantification of TDP43 relative amounts in chromatin and whole lysate co-IPs are indicated in the histograms. Mean+ SEM are indicated. Unpaired t test, two-tailed was done. Other details as in Fig 6 and S1 Fig. (TIF) [file pgen.1009260.s005.tif]
