# Supplementary material for: TDP-43 mutations link Amyotrophic Lateral Sclerosis with R-loop homeostasis and R loop-mediated DNA damage
Source: PLoS Genet. 2020 Dec 10;16(12):e1009260. doi: 10.1371/journal.pgen.1009260 (PMC7755276; doi:10.1371/journal.pgen.1009260)
Supplement: S1 Table — (DOCX) [file pgen.1009260.s006.docx]

**Giannini et al.**

**Supplemental Tables**

**S1 Table.**  **Oligonucleotides used in this study**.

| **Primers used for qPCR** | **Sequence 5’ to 3’** |
| --- | --- |
| *APOE* Fwd | GGGAGCCCTATAATTGGACAAGT |
| *APOE* Rev | CCCGACTGCGCTTCTCA |
| *RPL13A* Fwd | GCTTCCAGCACAGGACAGGTAT |
| *RPL13A* Rev | CACCCACTACCCGAGTTCAAG |
| *MIB2* Fwd | CTCTCCTTGTCTGGGGCTC |
| *MIB2* Rev | CTGCCTCCCTCACCTGTC |
| *WDR90* Fwd | GTGCCAGGCTGTATTGCTT |
| *WDR90* Rev | GGGAAATGCAGACGTGTCAT |
| *EGR1 Fwd* | GCCAAGTCCTCCCTCTCTACTG |
| *EGR1 Rev* | GGAAGTGGGCAGAAAGGATTG |
| *SNRPN* Fwd | TGCCAGGAAGCCAAATGAGT |
| *SNRPN* Rev | TCCCTCTTGGCAACATCCA |
| *HPRT Fwd* | GGACTAATTATGGACAGGACTG |
| *HPRT Rew* | TCCAGCAGGTCAGCAAAGAA |
| *TARDBP Fwd* | GGTGCAGGTCAAGAAAGAT |
| *TARDBP Rew* | GCTCATCTTGGCTTTGCTTAA |
