# Supplementary material for: TDP-43 mutations link Amyotrophic Lateral Sclerosis with R-loop homeostasis and R loop-mediated DNA damage
Source: PLoS Genet. 2020 Dec 10;16(12):e1009260. doi: 10.1371/journal.pgen.1009260 (PMC7755276; doi:10.1371/journal.pgen.1009260)
Supplement: Attachment — Submitted filename: PLoS Genet_Rebuttal.docx [file pgen.1009260.s008.docx]

**Rebuttal**

Reviewer's Responses to Questions

**Reviewer #1:**

In the manuscript “TDP-43 deficiency links Amyotrophic Lateral Sclerosis with R-loop homeostasis and R loop-mediated damage,” Giannini and co-authors explore the role of TDP-43 in the maintenance of genome integrity and try to dissect the mechanism of the neurodegenerative pathogenesis caused by the mutated version of the protein. They test the hypothesis that TDP-43 prevents the accumulation of DSB-inducing, deleterious R-loops and claim that an R-loop-dependent increase in DSB foci and Fanconi Anemia repair centers is caused by mis-localization of the mutated protein in transfected neuronal cells and in cell lines from ALS patients. Without a doubt, the findings reported in the manuscript are significant and worth further investigation; however, the conclusions drawn from the data are not sufficiently substantiated.

Thanks for the constructive comments and for the suggestions. After having attended them we believe that our conclusions are much strongly substantiated.

Major concerns.

1. The depletion of TDP-43 in HeLa cells by siRNA leads to a minor increase in S9.6 signal intensity (Figure 1A). Even though the increase is statistically significant, the biological relevance of such an increase is unclear without comparison to the changes in intensity after depletion of the known protein involved in R-loop processing.

The increase in S9.6 IF intensity upon TDP-43 depletion in Figure 1A graph is strongly significant (p-value<0.001, Mann-Whitney test, two-tailed), even though we agree that the increase was difficult to appreciate with the former graph scale. For this reason, we have adjusted the graph scale to facilitate visualization of this statistically significant difference.

To gain further insight into the biological relevance of such an increase, we have added and discussed data regarding relative increases in S9.6 IFs previously observed and published by depleting a wide range of factors involved in R loop-prevention (S1C Fig). According to the new data, depletion of TDP43 causes a relative increase in S9.6 median signal intensity per nucleus similar to that caused by other factors involved in mRNP biogenesis, such as THOC1 (S1C Fig).

This concern is exacerbated by the absence of an increase in DRIPqPCR product for the APOE gene in siTDP-43 treated cells (Figure 1B). Taking into account this marginal effect on R-loop formation on the one hand and the multiple functions of TDP-43 in mRNA processing and its interaction with DNA on the other, the increase in gammaH2AX and FANCD2 foci (Figure 2) could reflect consequences of TDP-43 depletion that are unrelated to R-loop processing. The decrease in foci in cells expressing RNaseH could also be an indirect consequence of the protein depletion.

We do not agree with this appreciation, which is not justified by our results. In any case, we have performed DRIP-qPCR analysis in more R loop-prone genes (WDR90, EGR1 and MIB2) and replicates that are included in the new version of Figure 1B. Now, 5 genes are shown to increase significantly R loop formation by DRIPqPCR in Figure 1B, confirming the IF results and strongly supporting a role for TDP43 preventing unscheduled R loop formation. Moreover, SNRPN control has been moved to Supplemental data (Supp. Figure 1D) as it is simply used as a negative control of the DRIP technique, since SNRPN is a poorly transcribed gene and R loop levels are expected to be very low at this gene. We show SNRPN together with RPL13A in S1D Fig for comparative purposes to show that it worked as a negative control.

In addition, we have included a new section on genome-wide data of TDP43 ChIP-seq and S9.6 DRIP-seq that support the global role of TDP43 in protein-coding genes (Fig 3).

We are certainly aware that TDP43 is involved in multiple mRNA processing mechanisms and it may lead to DNA damage through several mechanisms, but our study is focused on R loop-dependent DNA damage. The DNA damage marks studied (γH2AX and FANCD2 foci) decrease significantly by overexpressing RNase H1, a protein known to degrade the RNA moiety of RNA-DNA hybrids, confirming the R loop-dependency of such DNA damage. This methodology (RNase H1 overexpression) has been widely used in the literature to study R loop-dependency of DNA damage (Pérez-Calero, et al., 2020; Salas-Armenteros, et al., 2017). Certainly, our results do not exclude the possibility that TDP43 could also give rise to DNA damage through other mechanisms as DNA damage is significantly but not completely abolished by overexpressing RNase H1 (Figure 2). However, this is common to most cases in which suppression of DNA damage by RNH1 has been reported (reviewed in García-Muse and Aguilera, Cell 2019).

2. The statement from the abstract, “we prove that mislocalization of mutated TDP-43 (A382T) in transfected neuronal SH-SY5Y and lymphoblastoid cell lines (LCLs) from an ALS patient cause R-loop accumulation, and R loop-dependent increased DSBs and Fanconi Anemia repair centers” is misleading. Mis-localization of both proteins - mutant and wild type - leads to an increase in S9.6 intensity (Figure 3A), and the difference in S9.6 signal intensity between the cells with mutant and wild type proteins is not significant.

We appreciate this comment and agree that the message needed to be clarified and further supported. As mentioned before, TDP43 is involved in several mRNA processing steps and its alteration could be linked to an increase of different RNA structures, such as dsRNAs. It has also been reported that despite having a high affinity for DNA:RNA hybrids, S9.6 antibody also retain some affinity, though lower, for dsRNA. For this reason, we repeated the entire experiment presented in former Fig 3A (new Fig 4) but treating all samples with RNase III, a nuclease that degrades dsRNA, in order to gain specificity regarding DNA:RNA hybrid detection in these cell lines. As can be observed now in the new version of Figure 4A, S9.6 signal intensity per nucleus increase significantly when mutant forms of TDP43 are overexpressed. Thanks for helping us strengthening the message

It is well documented that overexpression of proteins can cause their mis-localization and misfunction. So, “significantly decreased” nuclear localization of the mutant protein in SHSY-TDP382 cells (decrease of less than 15%) compared to wild type SHSY-TDP+ cells (Supplementary Figure 1) can result from (slightly) different expression levels of each of the proteins in the corresponding cell lines. In other words, the somewhat increased deleterious consequences of expression of the mutant allele could be caused by a higher level of overexpression of the mutant protein, compared to the wild type allele, rather than the presence of a missense mutation. Unless the levels of TDP+ and TDP382 proteins are measured and found similar, the “mutation” effects could be attributed to the differential expression levels of TDP-43 alleles in the cells.

Performed as requested. We have added data regarding overexpression levels of TDP43 WT and TDP43 A382T mutant and. These data are shown in Supplemental Figure 2A-C and proves that TDP43 WT and A382T mutant forms are overexpressed to similar extents, excluding the possibility that the effects caused by the overexpression of the mutant form could be attributed to a major overexpression level in SHSY cell line. This study has been extended to a second TDP-43 mutation and the presence in nucleus versu cytoplasm have also been determined. Thank you.

3. The same critique applies to the experiments with the lymphoblastoid cells from the patients.

Compared to SHSY, LCLs come from patients and harbor the indicated mutations in the chromosomes. They have not been manipulated in any way to overexpress any form of TDP-43. For this reason, the same critique is not valid to these results.

In Pansarasa et al. 2018, lymphoblastoid cell lines from an healthy donor (Ctrl), an ALS patient carrying mutations on TARDBP (A382T, G294V) and a sporadic ALS patient, all used in this paper, were characterized. We showed the presence of cytoplasmic aggregates in TARDBP-mutated patients (Figure **A** from Pansarasa et al. 2018). Moreover , WB results (Figure B from from Pansarasa et al.2018) showed decreased TDP-43 protein level in all ALS patients compared to Ctrl. In the cytoplasm, we reported an increase in TDP-43 protein expression levels only in TARDBP-mutated patients compared to Ctrl, as can be seen in the accompanying figures.

4. The data in Figure 6 are neither quantified nor normalized. The quantity of the loading control proteins varies from lane to lane. Also, it is unclear how many times the experiment has been performed, so the discussion of the results of this experiment is mere speculation.

Data presented in Figure 6 aimed to show that TDP43 is detected by co-IP using S9.6 antibody, as expected given its multiple roles in mRNP processing, and that this co-IP is diminished in LCLs harboring A382T TDP43 mutation in accordance with IF results presented in Supplemental Figure 1. To clarify the message and to deal with comparable data between IF and co-IP, we have expressed TDP-43 nuclear content as total TDP43 nuclear signal intensity (new S2B Fig) and confirmed that nuclear area is not disturbed by overexpression of such proteins, excluding the possibility that differences in signal intensity could be attributed to changes in nuclear size. We also calculated the ratio TDP43/H3 co-IPed with S9.6 antibody in the chromatin fraction and the TDP43/GAPDH ratio for the whole lysate. It can be clearly observed now that nuclear/chromatin TDP43 content is significantly diminished by overexpressing a mutant form of TDP43 (A382T) or in LCLs carrying this mutation (new S2E Fig, Fig 6A and S4A Fig). In both cases, data comes now from 3 biological independent experiments. Thank you for making the message stronger.

5. In Figure 3A, the increase in S9.6 signal in the cells with overexpression of TDP+ cannot be attributed to R-loop formation because overexpression of RNaseH does not decrease the signal. Nevertheless, the authors make the following conclusion: “Therefore, we conclude that mislocalisation of TDP-43 also causes R-loop accumulation “(page 8). This statement contradicts the data. It is unclear whether the levels of overexpression of RNaseH were monitored during the experiments in different cell lines. One explanation of these data could be that the expression of RNaseH in the specific cell line was different (lower) from the others.

As mentioned above, experiments from Fig 3A have been repeated by treating all samples with RNase III, giving consistent results regarding RNase H1-dependency of the signal. This technique has been reportedly checked and is commonly used with reliable results (Pérez-Calero, et al., 2020; Salas-Armenteros, et al., 2017, García-Benítez et al, PNAS 2017). Nevertheless, to discard any bias potentially caused by different RNase H1 overexpression levels, we have quantified the percentage of transfected cells in IFs as control, and no significant changes were detected. This demonstrates that any signal difference observed after RNase H expression is not due to different RNase H1 expression levels between conditions.

6. Throughout the study, the only assay utilized for providing evidence for DNA damage or activation of DNA damage response is the detection of DNA repair foci. Additional, independent tests (such as measurement of homologous recombination or non-homologous end-joining in reporter systems, evaluation of checkpoint activation, assessment of sensitivity to DNA damaging agents) are necessary to address physiological consequences of TDP43 malfunctioning.

We agree that it would be also interesting to gain further insight on the consequences of TDP43 dysfunction in DNA repair, but this is not the aim of this work. Here, we aimed to establish a connection between TDP43 and R loop-dependency of the DNA damage arising when depleting the protein, and we simply use gammaH2AX and FANCD2 foci different DNA damage markers. In any case, even though the experiments proposed are of interest, those data will not add much on the way TDP3 protects from R loops.

Minor concerns.
1. There is no description of how overexpression of RNaseH is achieved in Figures 1-5. Reference #33, used for an explanation (Ginno et al., 2013), does not provide relevant information.

We have corrected this in Results. It was a mistake. The method is explained in M&M using the appropriate reference #61 (Cerritelli et al 2013). Thanks.

2. Since in Figures 3A and Supplemental Figure 1, the data presented as “signal per nucleus” and the differences in these signals are minor, it seems to be important to make sure that the area (size) of the nuclei are similar among cells transfected with different constructs.

S9.6 signal is presented in these figures as S9.6 mean nuclear signal per cell, so nuclear area does not affect S9.6 nuclear mean intensity. It would affect if we were using S9.6 nuclear signal summation per cell, but it is not the case. However, we have also quantified nuclear area in the different cell types to prove that even though this is not the case (S2F Fig). In any case western blot after fractionation has been added to support our conclusions (S2B Fig)

3. Flow cytometry plots in Figures 5B and C do not include the plot of S9.6 signal for the LCL-SALS cells (cells from sporadic ALS patient), making the data representation skewed.

We have added the required plots. The yellow peak is relative to S9.6 staining on SALS LCL compared to the blue peak representing the CTRL LCLs (Fig 6B)

**Reviewer #2:**

In this manuscript, Gianini et al. have investigated in detail the role played by TDP-43 in the generation of R-loops and RNA:DNA hybrids in neuronal and non-neuronal cell lines. In general, the manuscript contains interesting data for the field and several novel observations that contribute to a better understanding of the protein in disease. A few required additions and clarifications are as follows:

Thanks for the positive and constructive suggestions.

-The title “TDP-43 deficiency links Amyotrophic Lateral Sclerosis with R-loop homeostasis and R loop-mediated DNA damage” does not seem appropriate since the manuscript principally focuses on a disease-associated mutation, A382T. Therefore, it might be better to change it in “TDP-43 mutation links Amyotrophic Lateral Sclerosis with R-loop homeostasis and R loop-mediated DNA damage”.

Changed as requested.

In any case, by referring to deficiency of TDP43 we referred to dysfunction, regardless of whether it is caused by depletion though siRNA treatment (i.e. our experiments in HeLa) or by using patient lines carrying TDP43 mutations or overexpressing these mutated forms and causing a dominant negative effect, causes R lop-dependent DNA damage, thus linking ALS with R loop-dependent DNA damage.

- The order followed in the abstract is not the one respected in the results (ie. in the abstract the authors first write about mislocalization of mutated TDP-43 (A382T) in transfected neuronal SH-SY5Y and lymphoblastoid cell lines (LCLs) from an ALS patient cause R-loop accumulation and then a non-neuronal model of HeLa cells depleted of TDP-43. However, in the results they present first the depletion of TDP-43 in Hela cells and then the transfection of mutated TDP-43 in SHSY-5Y. Please be consistent in the order of the text.

We have modified it to make it consistent. Thanks

- In the work the authors used silenced TDP-43 in Hela cells but transfected mutated TDP-43 in SHSY-5Y. Please explain better the choice of the two cell lines and how they are connected.

LCLs (Lymphoblastoid Cell lines) are patient-derived cell lines, while SH-SY5Y are neuroblast-like cells usually used as in vitro models of neuronal function and differentiation. Given the neurological disease nature of ALS, most studies focusing on ALS are done in SH-SY5Y cells. However, HeLa cells are also widely used for functional analysis. For this reason, we started the study in HeLa cells by depleting TDP43 and checking whether it had an impact on R loop-dependent genome instability. Once established this connection, we extended the study to SH-SY5Y cells, a cell line often used as model of neuronal function, overexpressing mutated forms of TDP43 and finally to LCLs from patients carrying the mutation. This has been now better explained in the text at the beginning each cell type is introduced in the study in the Results section. Thanks

- In the LCLs mutated in TDP-43 the authors observe an increase of R-loops compared to CTL and SALS LCLs. RNAse H could revert partially the S9.6 increase signal. How can this partial decrease be explained?

The S9.6 antibody recognizes specifically DNA:RNA hybrids but it also possess some affinity for other forms of RNA, even though to a lesser extent, and overexpression of this RNH is widely used to confirm specificity of S9.6 on hybrids. In experiments involving S.6 antibody, overexpression of RNase H1 abrogates antibody signal when the signal is DNA:RNA hybrid-dependent. However, this suppression is in many cases partial, because either other RNA molecules are recognized, the ectopic expression of RNH may be limited in some cell lines, or because not all cells are transfected by the plasmid carrying eGFP. In this case, our results confirm an accumulation of R loops in LCLs from patients carrying the A382T mutation but exclude this possibility in LCLs from SALS patients, meaning that R loops accumulate in ALS patients caused by mutations in TDP43 but not in those were the disease is classified as sporadic (SALS). We believe that this is clear along the text.

- Experiments were run in SHSY5Y and in LCL carrying only one mutation A382T. It might be interesting to carry out at least part of the reported experiments in SHSY-5Y cells overexpressing TDP-43 with another mutation. These data would confirm whether the readouts observed with this mutation are specific or not.

Performed as requested. We appreciate this comment.

We have added data concerning another ALS-associated TDP43 mutation (G294V). As can be observed in Figure 4A, R loops also accumulate when this mutant form of TDP43 is overexpressed in SHSY-5Y cells, excluding the possibility that the observed phenotype was a defect restricted to A382T TDP43 mutation and pointing to TDP43 deficiency itself as the main cause of the observed R loop-dependent DNA damage. Thanks.

**Reviewer #3:**

The paper by Giannini et al focuses on understanding how deficiency of TDP-43 observed in ALS is connected with R-loop homeostasis and R-loop mediated DNA damage. Previous work from Livinsgton lab has demonstrated that TDP43 is involved in prevention/repair of transcription-associated DNA damage through R-loop-related mechanisms in neuronal cells (Hill et al PNAS 2016), therefore the novelty of this paper is partially compromised by this publication. One interesting aspect which the authors were trying to pursue is to uncover if TDP-43-disease-associated mutations which result in re-localisation of usually nuclear TDP43 to the cytoplasm has any effect on R-loop-mediated DNA damage. This is certainly an interesting question worth addressing. The authors pursued these questions in HeLa cells (where TDP43 is depleted, Fig 1-2), neuroblastoma cells (where TDP-43 wt or mutant is over-expressed, Fig 3-4) and lyphoblastoid cells (with patient mutations in endogenous TDP43, Fig 5-6). Using these model systems, the authors have shown that TDP43 deficiency results in accumulation of R-loops (by IF and DRIP-qPCR), DNA damage (γH2AX) and Fanconi Anemia repair centers. Unfortunately, the authors have not managed to take these initial findings, some of which were already reported in Hill et al PNAS 2016. (i.e. R-loop and DNA damage increase), into subsequent mechanistic investigations. For the reasons of the quality and amount of data presented in this paper, at this current stage the paper is not suitable for publication in PLoS Genetics.

Thanks for the nice summary of our contributions and suggestions. We strongly believe that the manuscript suitable for PLoS Genet, and now much more after attending the suggestions of the three referees.

Major comments:
1. The amount of data presented in this paper is minimal and not of a good quality which overall does not give me the confidence of the general paper conclusions. In particular the conclusions drawn from S9.6 IF analysis are rather ‘pushed’ and not visible from the images presented. For example, Fig 1A does not have a clear nuclear signal in any of the images shown (only nucleolar signal is visible). The differences between quantified signals are very small and represent only a background signal, in my opinion). The RNase H over-expression does not result in any visible change of the signal on the images. The positive controls (known factor which up-regulates R-loop level) are missing. The authors have not quantified nucleolar signal (are there any changes there?), though they have chosen to study gene encoding ribosomal protein in their DRIP analysis (see below).

All data presented in this manuscript have been subjected to the corresponding statistical analysis and significant differences are reported in the graphs. However, we agree that some example images required improvement and needed to be adjusted to cell population results reported in the corresponding graphs, as also suggested by referee 1. For this reason, we have adjusted settings in some of them to show a more accurate image of the IF and replacing some with others that fit more accurately to cell population results. Furthermore, as mentioned before, fold changes in S9.6 median increase caused by depletion of different factors involved also in R loop metabolism are provided (S1C Fig) for comparative purposes, and properly discussed in the text.

Finally, we do not intend to make a distinction between RNA polymerases, which is an interesting question to be addressed in the future. Given the repetitive nature of the rDNA, it makes difficult to get reliable data in those regions. We focused on the role of TDP43 during mRNP biogenesis to prevent R loop formation on protein-coding genes. We normally include ribosomal proteins because they are highly expressed, thus becoming more sensitive to R loop accumulation, since these genes are transcribed by RNAPII and are outside of the nucleolus.

Now, we have included a new section on genome-wide data of TDP43 ChIP-seq and S9.6 DRIP-seq that support the global role of TDP43 in protein-coding genes (Fig 3).

2. It is surprising to me that the only gene which shows statistically significant accumulation of R-loops is RPL13A, which is a ribosomal protein. I would like to see more comprehensive analysis of R-loop increase by DRIP-qPCR. Other multiple genes of different categories should be tested in different model systems.

Performed as requested. We have extended the DRIPq-PCR analysis to other RNA polymerase II-dependent genes (WDR90, EGR1, MIB2), as mentioned above. Currently, significant increases by DRIP-qPCR have been determined for 5 these 5 genes (Figure 1B).

3. Experiments in Fig 3-4 are carried out with neuroblastoma cells, where TDP-43 Wt or mutant (TDP382) are expressed on the endogenous background. The authors need to characterise this system a bit better, since the imaging in Fig S1 is not sufficient. They need to present Westen blot showing the level of expression of endogenous and over-expressed proteins in different cellular fractions (i.e. nucleus, cytoplasm) in all three cell lines. What is the level of endogenous protein contribution to all results observed? The authors have not really explained why do we observe a significant decrease of nuclear signal for TDP43 WT cells compared to control cells? These data are important for further interpretation of IF and DRIP results. All these experiments should have been performed on the background of depleted endogenous TDP-43 protein to have a clear interpretation.

We agree that additional controls would strengthen the results reported and would help interpreting them. Following these suggestions, we have added western blot, IF and flow cytometry data with their corresponding quantifications showing that expression levels remain similar when overexpressing a WT or a mutated form of TDP43 (S2A Fig). Changes are observed in the ratio between nuclear and cytoplasmic presence by IF and Western are noe included (S2B,E Fig)

Regarding the significant decrease of TDP43 nuclear signal when overexpressing a WT version of the protein, it has already been reported that overexpression of TDP-43 is detrimental to cells (Cannon et al., 2012; Heyburn and Moussa, 2016; Lu et al., 2016). Phospho-TDP43 aggregation and cytoplasmic inclusions have been reported when overexpressing TDP43 (Cannon et al., 2012). It is possible that simply overexpressing the WT form of the protein results in a partial quenching of TDP43 in the cytoplasm. This could explain why R loops are observed in some genes by DRIP simply by overexpressing the WT form of the protein. However, this effect is clearly and significantly enhanced when the A382T mutated form of the protein is overexpressed, as reported. G294 mutant is also an increased, but there is not a detectable reduction in the nuclear fraction, implying that in this case the effect is also due to a dysfunctional protein. Discussion on this topic has been added in the text and quantification of total nuclear TDP43 (endogenous+overexpressed) from IFs and Western blots are shown in S2 Fig. See also response to reviewer #1.

4. The authors have carried out IF in Lymphoblast cells from patients with TDP43 and ALS mutations. Similar to my comments for Figures 1A and 3A, the IF data are not convincing and show extremely small changes between cell lines studied. These cells derived from different people and are subject to patient –to patient genetic variation. Since the data are showing rather small changes, the authors should use multiple cell lines representing each category. Possibly using alternative approaches such as slot blot may provide more quantitative data than IF.

Thanks for this relevant comment. S9.6 and TDP43 IF performed in LCLs have been subjected to statistical analysis and statistically significant differences are reported. As mentioned before, we agree that some images needed to increase image quality or be replaced by better ones. For this reason, we have improved the images to provide a better visualization of the examples presented that fit better to the quantification results.

ALS is a rare disease which can occur sporadically, without any family history (90-95% of patients), while a small percentage of ALS cases are considered familial (5-10%). Patients carrying mutation on TARDBP constitute 3% of cases, so they are very rare. Therefore, it is quite difficult to find multiple cell lines representing each category.

5. Figure 6 contains results of S9.6 IP experiments in lymphoblast cells. The authors propose that they observe less TDP43 interaction with R-loops when TDP43 is mutated. This is not visible from the protein gel presented, and as far as I am concern represents a wishful thinking of the authors and not the result of a given experiment. There is absolutely minimal pull-down observed with TDP-43 in the nucleus, and mainly there is a pull down of TDP-35 which should be really cytoplasmic, and not in the chromatin fraction? Clearly these TDP-35 fragments are also IPed in the WT conditions – why is this happening? This experiment lacks RNase H control to show that interaction between TDP43 and R-loops is specific. Comments regarding stronger pull-down of TDP35 in TDP43 mutant in cytoplasm are not really substantiated since it is clear that this sample is over-loaded on the gel as can be seen from H3 and GAPDH westerns.

The aim of this experiment was to show that TDP43 can be co-IPed when using S9.6 antibody in wild-type cells, as both factors occur co-transcriptionally, and that this co-occurrence is disturbed when LCLs harbor A382T mutation consistently with TDP43 IF data. RNase H control in this experiment is not relevant for the purpose of this experiment as TDP43-R loop connection is already disturbed when LCLs are TDP43-mutated. On the contrary, a major accumulation of TDP43 is observed in the whole lysates co-IPing with S9.6, meaning that TDP43 accumulates in the cytoplasmic fraction together with S9.6 antibody targets, even though probably RNA structures such as dsRNA that can occur in the cytoplasm for which S9.6 also possess certain affinity. Nevertheless, this experiment has been moved to Supplemental data. Instead genome-wide data supporting a major presence of TDP43 at expressed genes were R loops also occur is now shown (Fig 3)

Finally, TDP35 is the cytoplasmic form, and we do not think that our study allows at this point to make conclusions about the role of this form in the context of R loop formation. We have changed the text to avoid confusion. Thanks

Minor comments:

1. On page 9 first paragraph, the authors say that ‘TDP-43 mutation causes an increase in DNA breaks derived from R-loop accumulation that promotes transcription-replication collisions. I think that this is an over-statement since it has not really been tested if accumulation of R-loops following TDP-43 depletion results in transcription-replication collisions.

It has been widely reported that R loops pose an obstacle for DNA replication fork progression and there is no reason to think the contrary when R loops accumulate like under TDP43 dysfunction conditions presented here. Our FANCD2 foci analysis supports this view. Indeed, a manuscript has just been accepted in J Cell Sci that supports this view by showing replication stress determined by DNA combing siTDP43 cells (ref. 57). This has been noted in the text.

2. The authors need to explain why SNRPN gene does not have DRIP signal – it is not expressed?

SNRPN is simply used as negative control of DRIP technique, since SNRPN is a poorly transcribed gene and R loop levels are expected to be very low at this gene. However, we show SNRPN together with RPL13A in Supp. Figure 1B for comparative purposes to show that it worked as a negative control. We have clarified this in the text. Thanks.

3. All the data in this paper can be grouped in 2-3 figures max (one figure for each cell model used).

We would prefer to present data distributed in different figures according to the message we want to transmit. It is important to note that dividing or not the figures will not shorten the paper, since panels will be still occupying the same space. This does not make a difference in a journal like PLoS Genet. IN any case, with the new experiments performed and new data included to attend the referees’ suggestions, it is not possible to reduce the number of Figures

4. The paper would benefit from additional English editing (i.e. - ‘significant’ on pages 6 and 11 should be spelled correctly.etc)

Corrected. Thanks.

5. Concluding model demonstrating TDP43 role in disease and R-loop biology would be helpful.

A model has been added as requested. Thanks
