# Supplementary material for: TDP-43 mutations link Amyotrophic Lateral Sclerosis with R-loop homeostasis and R loop-mediated DNA damage
Source: PLoS Genet. 2020 Dec 10;16(12):e1009260. doi: 10.1371/journal.pgen.1009260 (PMC7755276; doi:10.1371/journal.pgen.1009260)
Supplement: Attachment — Submitted filename: Revised manuscript by Gianini et al.docx [file pgen.1009260.s009.docx]

The revised manuscript by Gianini et al., “TDP-43 deficiency links Amyotrophic Lateral Sclerosis with R-loop homeostasis and R loop-mediated damage,” is much improved compared to the original version.

Most of the issues that caused concerns in the paper’s previous version regarding data interpretation and appropriate controls are addressed and resolved in the updated manuscript.

The added comparison of the consequences of TDP-43 depletion on R-loop formation (reported as changes in S9.6 fluorescence intensity) to the measurements already published in the studies of the contribution of proteins directly involved in R-loop processing (S1C Fig), as well as DRIP-seq of additional R-loop - prone targets, allows appreciation of the biological relevance of relatively minor changes in fluorescence levels.

The data from the new experiments involving treatment of the cells with RNAse III for the removal of dsRNA (Figure 4A) eliminate the ambiguity of the results reported in the first version of the manuscript and validate the statement, “we prove that mislocalization of mutated TDP-43 (A382T) in transfected neuronal SH-SY5Y and lymphoblastoid cell lines (LCLs) from an ALS patient cause R-loop accumulation…”

Measurements of the expression levels of the wild type and mutant constructs (reported in S2A and C Figures) strengthen the conclusion that indeed mislocalization, rather than an increased level of the mutant protein, causes accumulation of R-loops.

Several points still require the authors’ attention.

1. Even though the new genome-wide analysis of TDP-43 localization (Figure 3) does not provide direct evidence of the contribution of TDP-43 to R-loop processing but instead suggests that TDP-43 binds to highly expressed loci in DNA, these data offer new insights into TDP-43 functions. Nevertheless, the statement “…TDP-43 RBP controls transcription at expressed genes that are prone to accumulate R loops...” is not substantiated by the data and should be toned down.

2. Since statistically significant “reduction in nuclear fraction” of endogenous TDP-43 was NOT observed either following expression of SH-TDP+, or SH-TDP382, it is formally incorrect to attribute an absence of a reduction in SH-TDP294 to any phenotype (S2BFig). (“In the case of SH-TDP294, a reduction in the nuclear fraction was not observed in the western blot, therefore implying that protein dysfunction can be causative of the phenotype.”, p. 8).

3. The model in Figure 6C must be adjusted to accommodate the data showing that a decrease (compared to control) in nuclear localization of overexpressed wild type TDP-43 (2SE Figure, SH-TDP+ vs. SH-SY5Y) does not cause an increase in S9.6 intensity (Figure 4A).

4. The spreadsheets with the raw data reflected in graphs and figures are not provided.
