# Supplementary material for: TDP-43 mutations link Amyotrophic Lateral Sclerosis with R-loop homeostasis and R loop-mediated DNA damage
Source: PLoS Genet. 2020 Dec 10;16(12):e1009260. doi: 10.1371/journal.pgen.1009260 (PMC7755276; doi:10.1371/journal.pgen.1009260)
Supplement: Attachment — Submitted filename: Giannini et al_Response to reviewers_R2.docx [file pgen.1009260.s010.docx]

Reviewer's Responses to Questions

**Comments to the Authors:**
The review is shown below. Answers in blue.

Reviewer #1: The revised manuscript by Gianini et al., “TDP-43 deficiency links Amyotrophic Lateral Sclerosis with R-loop homeostasis and R loop-mediated damage,” is much improved compared to the original version.

Most of the issues that caused concerns in the paper’s previous version regarding data interpretation and appropriate controls are addressed and resolved in the updated manuscript.
The added comparison of the consequences of TDP-43 depletion on R-loop formation (reported as changes in S9.6 fluorescence intensity) to the measurements already published in the studies of the contribution of proteins directly involved in R-loop processing (S1C Fig), as well as DRIP-seq of additional R-loop - prone targets, allows appreciation of the biological relevance of relatively minor changes in fluorescence levels.

The data from the new experiments involving treatment of the cells with RNAse III for the removal of dsRNA (Figure 4A) eliminate the ambiguity of the results reported in the first version of the manuscript and validate the statement, “we prove that mislocalization of mutated TDP-43 (A382T) in transfected neuronal SH-SY5Y and lymphoblastoid cell lines (LCLs) from an ALS patient cause R-loop accumulation…”

Measurements of the expression levels of the wild type and mutant constructs (reported in S2A and C Figures) strengthen the conclusion that indeed mislocalization, rather than an increased level of the mutant protein, causes accumulation of R-loops.

We appreciate very much the positive comments. Thanks

Several points still require the authors’ attention.
1. Even though the new genome-wide analysis of TDP-43 localization (Figure 3) does not provide direct evidence of the contribution of TDP-43 to R-loop processing but instead suggests that TDP-43 binds to highly expressed loci in DNA, these data offer new insights into TDP-43 functions. Nevertheless, the statement “…TDP-43 RBP controls transcription at expressed genes that are prone to accumulate R loops...” is not substantiated by the data and should be toned down.

We have removed that statement. Instead, we have added: *“These results support that the TDP-43 RBP is present at expressed genes that are prone to accumulate R loops, where it might participate in mRNP biogenesis, similarly as proposed for other co-transcriptional RNA binding factors [8]. A remaining is which specific role TDP-43 may play during transcription of those genes.”*

2. Since statistically significant “reduction in nuclear fraction” of endogenous TDP-43 was NOT observed either following expression of SH-TDP+, or SH-TDP382, it is formally incorrect to attribute an absence of a reduction in SH-TDP294 to any phenotype (S2BFig). (“In the case of SH-TDP294, a reduction in the nuclear fraction was not observed in the western blot, therefore implying that protein dysfunction can be causative of the phenotype.”, p. 8).

We have changed this message to: “*In the case of SH-TDP294, the ratio between nuclear and non-nuclear signal did not show the same tendency, raising the possibility that protein dysfunction can be causative of the phenotype”*

3. The model in Figure 6C must be adjusted to accommodate the data showing that a decrease (compared to control) in nuclear localization of overexpressed wild type TDP-43 (2SE Figure, SH-TDP+ vs. SH-SY5Y) does not cause an increase in S9.6 intensity (Figure 4A).

We have modified the model accordingly. We agree that references to high and low nuclear TDP43 content in the model could be misleading, as argued by the referee. Now, the model fits more precisely to data obtained in this study. In this new version, two arrows emerge from the nucleus of a representative cell indicating two types of conditions, Functional TDP43 vs Dysfunctional TDP43. First, when TDP43 is functional (i.e. WT or o/e TDP43 WT) R loop-dependent DNA damage is prevented and genome integrity is preserved. However, under dysfunctional TDP43 conditions (i.e. A382T or G294V TDP43 mutations) R loops and R loop-dependent DNA damage arise threatening genome stability and leading to ALS.

4. The spreadsheets with the raw data reflected in graphs and figures are not provided.

It has now been included. Thanks

Reviewer #2: The authors have provided several new data and have addressed in detail most of the queries raised by reviewers.

Thanks very much for the positive reception of the revised manuscript

Reviewer #3: The authors have made an effort to improve this manuscript including additional genes for DRIP-qPCR analysis (as requested in my comment 2) and some modest visual improvement for S9.6 IF.

Thanks for your positive reception of the revised manuscript

Related to previous comment 1:
To address my concern related to small changes in IF signal in the nucleus associated with Pol II genes, the authors introduced genomic data from publicly available Encode and previously published data, showing a correlation between TDP-43 binding and R-loops in expressed genes under physiological conditions in K562 cells (fig 3). Based on these datasets TDP-43 is mainly enriched at the 5’end /TSS of the expressed genes, while R-loops can be found in multiple locations (as shown by the DRIP-qPCR examples in the paper, where TDP-43-sensitive R-loops are enriched across various genomic locations). However, these new genomic data in Fig 3 does not clarify what happens with this correlation when TDP-43 is depleted? Do R-loops accumulate only at the locations where TDP-43 is bound which would support the direct effects of TDP-43 on the regulation of these R-loops? The authors have not demonstrated if they see a specific enrichment of TDP-43 binding at R-loops of their tested genes by DRIP-qPCR supporting the specificity and direct effects of TDP-43 on these R-loops.

We realize that our work does not cover all questions raised in this point, which are certainly of interest, but they are beyond the scope of our study. However, the questions raised seems to be the result of a premise that at no point we are intending to establish in our study. At no moment we imply that TDP-43 has any specific role at R loops either by attaching to them or resolving them. As shown by other mRNA processing factors, a paradigm of which is the THO complex, we argue that it is the suboptimal action of TDP43 as an RBP during transcription what is linked to the appearance of R loops. The specific function of TDP-43 at this level is unknown, but we have no evidence to suggest that TDP-43 binds to R loops or is able to resolve them. These are questions that would have to be resolved in the future: given our yet unresolved question about how other RBPs like THO, SRS1 and a number of DDX proteins protecting from R loops. We have tried to make this message clearer in Discussion. We included in page 13: *“As shown by other mRNA processing factors, a paradigm of which is the THO complex, we propose that it is the suboptimal action of TDP43 as an RBP during transcription what is linked to the appearance of R loops. However, further research is required to completely understand how RBPs and a number of DDX proteins protect from R loops.”*

Related to previous comment 4:
Unfortunately the authors have failed to introduce additional patient cell lines to verify the effects of R-loop accumulation in patients with different genetic backgrounds. Therefore, in my opinion, providing a direct evidence that TDP-43 binds at the regions of R-loops affected following its depletion (as discussed above) is crucial.

We do not share this view. We added in the revised manuscript additional data on R loop accumulation upon overexpression of another TDP43 mutation (G294V) in SHSY-5Y cells (Fig. 4 and S3). Moreover, we also show data directly in ALS patient lymphoblastoid cell lines (LCLs) carrying A382T TDP43 mutation and SALS patient LCLs to show that not all ALS-associated genomic backgrounds result in R loop accumulation (Fig. 6 and S4,5), as this only occurs when TDP43 is perturbed. We agree that an extended study using multiple genetic backgrounds will enrich our knowledge on the impact of TDP-43 depletion, but this is not the purpose of this study. Once more the referee insists in the idea that TDP-43 binds to R loops, which as explained in the previous point neither we are proposing nor our data suggests it.
